# Expression of STAT3 and hypoxia markers in long-term surviving malignant glioma patients

**DOI:** 10.1186/s12885-024-12221-w

**Published:** 2024-04-23

**Authors:** Katerina Dvorakova, Veronika Skarkova, Barbora Vitovcova, Jiri Soukup, Hana Vosmikova, Zuzana Pleskacova, Adam Skarka, Michael Christian Bartos, Petr Krupa, Petra Kasparova, Jiri Petera, Emil Rudolf

**Affiliations:** 1 Department of Medical Biology and Genetics, Faculty of Medicine in Hradec Kralove, Charles University, Hradec Kralove, Czech Republic; 2 The Fingerland Department of Pathology, Faculty of Medicine n Hradec Kralove, Charles University, University Hospital Hradec Kralove, Hradec Kralove, Czech Republic; 3 Department of Oncology and Radiotherapy, Faculty of Medicine in Hradec Kralove, Charles University, University Hospital Hradec Kralove, Hradec Kralove, Czech Republic; 4 Department of Chemistry, Faculty of Sciences, University of Hradec Kralove, Hradec Kralove, Czech Republic; 5 Department of Neurosurgery, Faculty of Medicine in Hradec Kralove, University Hospital Hradec Kralove, Hradec Kralove, Czech Republic; 6 Department of Pathology, Military University Hospital Prague, Prague, Czech Republic; 7 Department of Pathology, First Faculty of Medicine, Charles University, General University Hospital in Prague, Prague, Czech Republic

**Keywords:** Glioblastoma, Hypoxia, Long-surviving patients, STAT3, Temozolomide, Primocultures

## Abstract

**Background:**

Glioblastoma is a malignant and aggressive type of central nevous system malignancy characterized by many distinct biological features including extensive hypoxia. Hypoxia in glioblatoma associates with complex signaling patterns including activation of several pathways such as MAPK, PI3K-AKT/mTOR and IL-6/JAK/STAT3 with the master regulator HIF-1, which in turn drive particular tumor behaviors determining, in the end, treatment outcomes and patients fate. Thus, the present study was designed to investigate the expression of selected hypoxia related factors including STAT3 in a small set of long-term surviving glioma patients.

**Methods:**

The expression of selected hypoxia related factors including STAT3 was evaluated in a time series of formalin fixed paraffin embedded and cryopreserved glioma samples from repeatedly resected patients. In addition, comparative studies were also conducted on primary glioma cells derived from original patient samples, stabilized glioma cell lines and tumor-xenograft mice model. Obtained data were correlated with clinical findings too.

**Results:**

Glioblastoma samples of the analyzed patients displayed heterogeneity in the expression of hypoxia- related and EMT markers with most interesting trend being observed in pSTAT3. This heterogeneity was subsequently confirmed in other employed models (primocultures derived from glioblastoma tissue resections, cryopreserved tumor specimens, stabilized glioblastoma cell line in vitro and in vivo) and concerned, in particular, STAT3 expression which remained stable. In addition, subsequent studies on the role of STAT3 in the context of glioblastoma hypoxia demonstrated opposing effects of its deletion on cell viability as well as the expression of hypoxia and EMT markers.

**Conclusions:**

Our results suport the importance of STAT3 expression and activity in the context of hypoxia in malignant glioblastoma long-term surviving glioma patients while emphasizing heterogeneity of biological outcomes in varying employed tumor models.

**Supplementary Information:**

The online version contains supplementary material available at 10.1186/s12885-024-12221-w.

## Introduction

Life expectancy of glioblastoma patients (e.g. beyond average 14 to 16 months since primary diagnosis) remains very low due to tumor aggressiveness and resistance to therapy associated with high recurrence rate [1]. Still, there are glioblastoma patients who live longer than 3 years and are hence classified as long-term survivors (LTS). Many efforts have been made to uncover concrete reasons that would define this exceptional group, however, without clear outcomes. Although select clinical characteristics as well as molecular features have been found useful in stratifying patients and predicting their fate, no single biomarker, subclinical signature or even genetic or epigenetic profile proved to be universally valid for glioblastoma LTS [2]. It is thus of paramount importance to identify concrete features or factors whose activities ultimately define behavior of this tumor and predict survival of patients. In this context, one of the promising and currently studied signature features of glioblastoma is hypoxia [3].

Glioblastoma represents a type of malignancy with very pronounced hypoxia, i.e., low oxygen tension, which is histopathologically characterized by several hypoxia-related features such as necrotic foci, pseudopalisades, and microvascular hyperplasia. These features are thought to originate from rapid proliferation of tumor cells that outgrow their blood supply, resulting in inadequate oxygen delivery. Additionally, highly vascularized malignant gliomas develop the abnormal vasculature often lacking in the structure and functionality necessary for efficient oxygen transport which further contributes to hypoxia within the tumor [4, 5].

Hypoxia in glioblastoma has pleiotropic effects influencing many biological aspects of its existence. Firstly, it triggers a complex intracellular signaling milieu comprising activities of hypoxia-inducible factors (HIFs), phosphoinositide 3-kinase-AKT kinase/mammalian target of rapamycin (PI3K-AKT/mTOR), mitogen activated protein kinases (MAPK), nuclear factor kappa B (NF-κB) and signal transducer and activator of transcription 3 (STAT3) pathways that promote tumor cells survival, aggressiveness, invasion and resistance to therapy [6, 7]. The key role here is attributed to HIFs, with HIF-1α and HIF-2α being the major HIF isoforms mediating the positive HIF-dependent signaling [8]. Both of them participate in many processes related with glioblastoma, such as angiogenesis, hypoxia-mediated apoptosis, genetic alterations or immune evasion [9]. Secondly, hypoxia can also induce genetic changes via HIF-1α in glioma cells, leading to the activation of genes involved in tumor growth, invasion, and resistance to therapy [10, 11]. Moreover, hypoxia within glioblastoma can impair the immune response against tumor cells including the recruitment of immune-suppressive cells, such as regulatory T cells and myeloid-derived suppressor cells, while also reducing the activity of cytotoxic immune cells [12]. Thirdly, hypoxia-induced changes in the tumor microenvironment can render gliomas resistant to various treatments, including radiation therapy and certain chemotherapeutic agents [13] which correlate with predicted survival of patients [14].

Concerning the prominent role of hypoxia in glioblastoma biology, our experimental study has been designed to investigate the expression of select hypoxia and epithelial-to-mesenchymal (EMT)-related markers in a small group of LTS glioblastoms patients repeatedly treated in our university hospital. Specifically, we aimed to study and compare expressions of these markers in a time series of resected tumor samples and cell cultures derived from them. The main rationale of such an approach was based on the recognized role of hypoxia in pathogenesis of glioblastoma as well as the missing evidence concerning the involvement of hypoxia in individually recurring tumors and their behavior.

## Materials and methods

### Clinical samples

Clinical tumor samples were obtained from eight patients who repeatedly underwent surgery for malignant glioma at University Hospital Hradec Kralove. The study was approved by local ethics committee (Reference No. 201,906 S26P) and patients gave their written consent. All samples included in the study differed in tumor stage and grade and represent a differing number of repeated resections. The material sampled (amount and quality) for RNA analysis of hypoxia markers as well as cell culture derivation varied based on the particular specimen.

The cell derivation procedure and further manipulation with cell cultures was described previously [15]. In this study, three glioma cell primocultures were prepared in sufficient quality and quantity required for further analyses.

Clinical data pertinent to each case were retrospectively or prospectively reviewed including surgical reports, radiological images, histological parameters and therapeutic protocol. Tumor size (before surgery) was evaluated by neuroradiologist and radiation therapist following Magnetic Resonance Imaging (MRI). It was performed by 3 Tesla magnet using a cross-sectional 2D method with the product of the largest perpendicular diameters on T1-weighted contrast-enhanced. Other parameters such as tumor location, survival, response to radiotherapy and TMZ treatment, electrocortigraphy (ECOG), among others were evaluated and correlated too.

### Immunohistochemistry and tissue microarray (TMA) construction

Paraffin-embedded specimens, cryopreserved samples and native (non-fixed) tumor tissue samples were retrieved from and handled by Fingerland Department of Pathology. In case of native tumor samples, a minor part was cryopreserved and most of tumor tissue was fixed in 4% buffered formalin solution and paraffin embedded. 1 μm thick sections were cut and stained with hematoxylin-eosin to confirm the diagnosis and select appropriate areas for additional analyses. A TMA was constructed from tumor samples, using TMA Master II system (3DHISTECH Ltd., Budapest, Hungary). For each case, 2-mm-thick sections were cut and routine H&E staining and immunohistochemical studies were performed. The list of antibodies and relevant details of immunohistochemical protocols are summarized in Supplementary Table 1. Heat induced epitope retrieval (HIER) was used for antigen retrieval, employing different pH according to the antibody. For EGFR, proteolytic pretreatment step was used. The sections staining was carried out on Benchmark Ultra stainer manufactured (Ventana/Roche, Tucson, AZ, USA) using either Ventana ultraView Universal DAB detection kit or Ventana OptiView DAB IHC detection kit: both methods use avidin-biotin complex method with horseradish peroxidase as an enzyme and DAB (3,3’-diaminobenzidine) as chromogen. Agilent/Dako Autostainer 48 or Dako Omnis (Agilent, Santa Clara, CA, USA), with EnVision Flex detection kit was used for IDH1 R132H, CD57, ZEB2, TWIST, and HIF1a. For HIF1a and HIF1b, whole sections (WS) from tissue core donor blocks were used. The slides were subsequently counterstained with hematoxylin.

### Mutation analysis

DNA from glioma cells was extracted using the commercial DNA Sample Preparation Kit (Roche, Basel, Switzerland). Mutation analysis was performed by multi parallel sequencing (NGS) using the hybrid-capture-based target enrichment. A custom KAPA HyperChoice MAX Library (Roche) for enrichment of the coding and 30 bp upstream and downstream overlaps of selected panel of genes (TP53, EGFR, IDH1, IDH2, PTEN, PIK3CA) was used. Paired-end cluster generation and sequencing was performed by NGS system Illumina MiniSeq. Sequencing data analysis were performed by NextGENe software (Softgenetics) and Varsome Clinical Platform. MGMT methylation analysis was performed on DNA in FFPE using DNA Sample Preparation Kit (Roche). Bisulfite conversion of isolated DNA was performed using the EZ DNA Methylation-Gold Kit (Zymo Research). Detection and quantification of the hypermethylation status of the O (6)-methylguanine-DNA methyltransferase (MGMT) promoter was performed by the methylation-specific real-time PCR method using the CE-IVD marked geneMAP MGMT Methylation Analysis Kit (GenmarkSalgik).

### Athymic nude mouse model

Female Foxn1-nu athymic immunodeficient mice weighing 27–30 g were purchased from Velaz, Czech Republic. They were given a standard sterilized diet and water ad libitum. Cells suspensions intended for implantations were obtained from cultivated cells. Prepared 100 µl of cell suspension (1mil. cells/application) was injected subcutaneously into each 6 weeks old Foxn-1nu female mice on the right and left side of the back. The administration of treatment (TMZ) began two weeks after implantation (daily from day 15th to day 28th) as based on the approved project plan (ethical committee, project number: MSMT- 18,525/2021-3 attached as supplementary Fig. 4). TMZ was dosed orally (maximum volume 100 µl) at the therapeutic range of 0.9 mg/kg. Each experimental group consisted of 2–3 mice. The size of the tumors and health condition of mice were periodically checked. On the day of last application of TMZ, twenty minutes after treatment, mice were anesthetized with isoflurane, sacrificed and tumors were weighed and preserved until further analysis (in formalin at room temperature for IHC analysis, in Trisol at -80 °C for RT-PCR analysis and in lysis buffer at -20 °C for western blot analysis). Heart, liver, brain and plasma were also collected and stored at -80 °C before MS analysis.

### Cell line

Human malignant glioma cell line U87MG was purchased from ATCC (LGC Standards, Poland). Fresh cells from frozen batch were used for every set of experiments (lasted 3–9 weeks). Cultures were grown in EMEM supplemented with 10% FBS and 0.5% penicillin/streptomycin and cells were maintained in incubators with a humidified atmosphere containing 5% CO_2_ at 37 °C. The absence of mycoplasma contamination was periodically checked.

### Crispr/Cas STAT3 knockout cell model

Glioma cells U87MG grown to 50–70% confluence were transfected with transfection mixture (gRNA vectors in Opti-MEM I, the donor DNA and Turbofectin 8.0 - the ratios of 3:1 for Turbofectin: DNA) as based on manufacturer’s protocol (STAT3 Human Gene Knockout Kit (CRISPR), CAT#: KN204922, Origene). Cultures were split every 3 days (2–4 times in total) to dilute out cells containing non-integrated donor DNA. Then, puromycin selection was performed; cells of split P5 were grown directly in the puromycin containing complete media (the range of puromycin concentrations was 1 to 10 µg/ml) and medium was changed every 2–3 days. The puromycin resistant cells (approx. P10 split cells) were analyzed for genome editing, WB was used to measure gene knockdown and genomic PCR was carried out to verify the integration of the functional cassette. With primer pair of 5 F and 3R, both alleles of donor inserted and non-edited/indel were amplified. Thus established cell line was labeled as U87MG STAT3 KO.

### Proliferation

The inhibitory effect of chemotherapeutic TMZ at various concentrations on viability of U87MG and its STAT3 KO variant as well as viability of primocultures was evaluated by WST-1 assay for 48 h. Principle of this colorimetric test is based on the cleavage of the tetrazolium salt to colored formazan by mitochondrial dehydrogenases in viable cells. At the end of tested interval, cells were rinsed with PBS and WST-1 solution (diluted according to manufacturer’s recommendations) was added to each well for further 2 h. Quantification of mitochondrial enzymes activity was carried out at 450 nm with 650 nm of reference wavelength by Tecan Infinite M200 spectrophotometer (Tecan, Switzerland).

### RNA extraction, cDNA synthesis, primer design, quantitative real-time RT-PCR

Total RNA was isolated from glioma primocultures and both variants of U87MG– STAT3 expressed and STAT3 KO variant - using Direct-zol RNA MiniPrep kit according to the manufacturer’s instructions (ZymoResearch, Irvine, CA, USA). Tumors from cryopreserved tissues and excised from mice (both variants of U87MG tumors) were homogenized using Tissue lyzer (2 cycles − 25 vibration/s; 4 °C; 1 min; Qiagen, USA) in TriReagent. RNA concentration and its purity were measured using NanoDrop 2000 (Thermo Fisher Scientific). All samples had absorption ratio A260/A280 greater than 1.8. RNA integrity number (RIN) was determined using Agilent 2100 Bioanalyzer and cell line samples with RIN higher than 9.0, resp. tissue samples with RIN higher than 8.0 were used for further analysis. First strand cDNA synthesis and qPCR analysis were performed in LightCycler® 96 Instrument (Roche Life Science) as described in [16]. Primers were designed manually, and their sequences are attached as a supplementary file (Supplementary Tables 2 - primer sequences). Calculations were based on delta-delta Cq method [17] and expressed as fold change of the treated groups relative to the control. Beta-2-microglobulin (B2M) or TATA box binding protein (TBP) were used as reference genes for mRNA analysis.

### LC-MS analysis

Frozen tissues were homogenized in 4 volumes of cold PBS (w/v) using Fastprep-24 5G sample disruption instrument. Thawed plasma or homogenized tissues in the volume of 100 µl, was mixed with the same volume of methanol and acetonitrile, vortexed for 15 min and centrifuged at 14,000 g for 3 min. Supernatant was then filtered through 0.22 μm PTFE syringe filter into the vial and measured.

Detection of TMZ and its metabolites AIC and MTIC was performed on the Agilent 1290 Infinity II UHPLC system coupled to the Agilent 6470 QqQ mass spectrometer. Chromatographic conditions were maintained at gradient elution of 0.4 ml/min by 0.1% formic acid in water and methanol (0-0.5 95:5, 0.5-3.0 gradient to 5:95, 3.0–4.0 5:95, 4.0–5.0 95:5), thermostated autosampler set to 15 °C and column thermostat equipped with the Zorbax Eclipse plus RRHD C18 2.1 × 50 mm, 1.8 μm (PN 959757-902) column kept to 30 °C. MS source parameters were set to the following: drying gas 200 °C at 2 l/min, sheath gas 400 °C at 12 l/min, nebulizer pressure 25 psi, capillary voltage 2500 V and nozzle voltage 0 V. TMZ transitions of [M + H] + ions m/z were detected with setting of dwell time 50 ms, cell accelerator 4 V, fragmentor 88 V for 195→138 and 55 (collision energy– CE 8 and 28 V). MTIC transitions of [M + H] + ions m/z were detected with setting of dwell time 50 ms, cell accelerator 4 V, fragmentor 88 V for 169→109 and 43 (CE 20 and 40 V). AIC transitions of [M + H] + ions m/z were detected with setting of dwell time 50 ms, cell accelerator 4 V, fragmentor 88 V for 127→110, 82 and 55 (CE 20 V, 20 V and 40 V).

### Morphology

The TMA was evaluated for presence of the tumor tissue. Only TMA cases with at least one representative core were included for further analyses. The immunohistochemistry results were first digitalised using Leica Aperio AT2 slide scanner (Leica Biosystems, Buffalo Grove, IL, USA) and then evaluated with Aperio ImageScope software (Leica Biosystems, Buffalo Grove, IL, USA). The assessment of immunohistochemistry was performed by an experienced neuropathologist (JS). Percentage of positive cells and most prevalent staining intensity (1– weak, 2– moderate, 3– strong) was noted. Average percentage of positive cells and modified H score (mHS = percentage * intensity) was used for the analysis as reported previously [18]. Whole sections were analyzed using the same approach.

### Fluorescence microscopy

U87MG and U87MG-STAT-KO glioblastoma cells were fixed with 2% paraformaldehyde (20 min, 25 °C), rinsed with PBS permeabilized, and blocked with 1% Triton X and 5% BSA in PBS (30 min, room temperature). The cells were incubated with a primary antibody against STAT3 (D3Z2G® Rabbit mAb, Cell Signaling) at 4 °C overnight. Then, the cells were washed three times with cold PBS (5 min, 25 °C) and were incubated for additional 1 h (room temperature) with Alexa Fluor 488-labelled anti-rabbit antibody. Thereafter, the cells were rinsed three times with PBS and labelled with DAPI (10 µg/mL). The specimens were mounted into the Prolong Gold mounting medium (Invitrogen-Molecular Probes, Inc., Carlsbad, California, CA, USA) and examined using fluorescence microscopy technique (Nikon Eclipse E 400 (Nikon Corporation, Kanagawa, Japan)). The results were analyzed using LUCIA DI Image Analysis System LIM 4.2 (Laboratory Imaging Ltd., Prague, Czech Republic). All the samples were tested in duplicates in three independent experiments.

### Statistics

Data in all tests used are expressed as an average ± SD from at least two experiments. The concentration of chemotherapeutic TMZ causing a 50% decrease of cell viability (IC50 value) was determined by Graph Pad Prism 7.0. Statistical analysis of the data from RT-PCR analysis, proliferation WST-1 assay and LC-MS analysis was carried out using TWO-WAY analysis of variance (ANOVA) followed by Sidak’s multiple comparison test significant at level of p˂0.05. Other data analysis was done with GraphPad Prism 7.0.

## Results

### Clinical characteristics

The case set of eight patients (four women and four men) with repeatedly histopathologicaly confirmed malignant glioma was retrospectively analysed. The first patient designated as Le was used as control patient, i.e. the one subjected to surgery but without anticancer treatment - radiotherapy or chemotherapy at the request of the patient. Other patients from the case set underwent surgery, radiotherapy and chemotherapy as based on a similar therapeutic protocol (see Table 1). In all patients the diagnosis of glioblastoma - grade IV glioma was histopathologically confirmed before first surgical resection. Most evaluated patients were in the age group of 60 years plus, just two patients were under 40 years. Median survival of the patients was 40 months since the initial diagnosis. The size of tumors was evaluated before surgery and compared between individual patients and individual resections, with no significant relationships found. Increased size of tumors was observed in three patients labeled as Ku, Do and Bi.

**Table 1:** An overview of clinical parameters in a set of patients with repeatedly resected gliomas.TU size (mm) = tumor size before surgery; RT dose = radiotherapy dose

sample	resection	age	histology dg.	Survival (months)	sex	RT dose	TMZ dose concomitant	TMZ dose adjuvant/number of cycles	TU size (mm)	TU location	IDH1 status
**Le**	1st	57	giant-cell glioblastoma	49	F	NO	NO	NO	50	parietal	wt
	2nd	58	dtto + postsurgical changes						16	parietal	wt
	3rd	59	dtto						40	parietal	wt
	4th	60	dtto						34	parietal	wt
**Pu**	1st	61	prim.gbm	44	M	60 Gy	160	400/6	20	temporal	wt
	2nd	62	gbm perzistence, postsurgical changes			13 Gy (gamma knife)			15	temporal	wt
	3rd	64	postsurgical changes, gbm infiltration						16	temporal	wt
**Ku**	1st	26	prim.gbm	55	M	60 Gy	-	-	37	tempoparietal	mt
	2nd	28	gbm reccurence, postsurgical changes						54	tempoparietal	mt
**Do**	1st	58	prim.gbm	31	F	60 Gy	140	300/12	25	frontal	wt
	2nd	61	reccurence						35	frontal	wt
**Jo**	1st	63	prim.gbm	36	F	60 Gy	140	NO	36	parietal	wt
	2nd	65	reccurence, postsurgical changes			12 Gy (gamma knife			36	parietal	wt
**Du**	1st	62	prim.gbm	34	M	60 Gy	140	300/6	53	frontal	wt
	2nd	64	gbm persistance						48	frontal	wt
**Bi**	1st	67	prim.gbm	24	F	60 Gy	140	NO	38	temporal	wt
	2nd	69	gbm reccurence						53	temporal	wt
**Na**	1st	38	prim.gbm	48	M	60 Gy	140	400/6	72	parietal	mt
	2nd	41	gbm reccurence			24 Gy (gamma knife)			34	parietal	mt

### IHC analysis of tumor samples

**Fig. 1:**
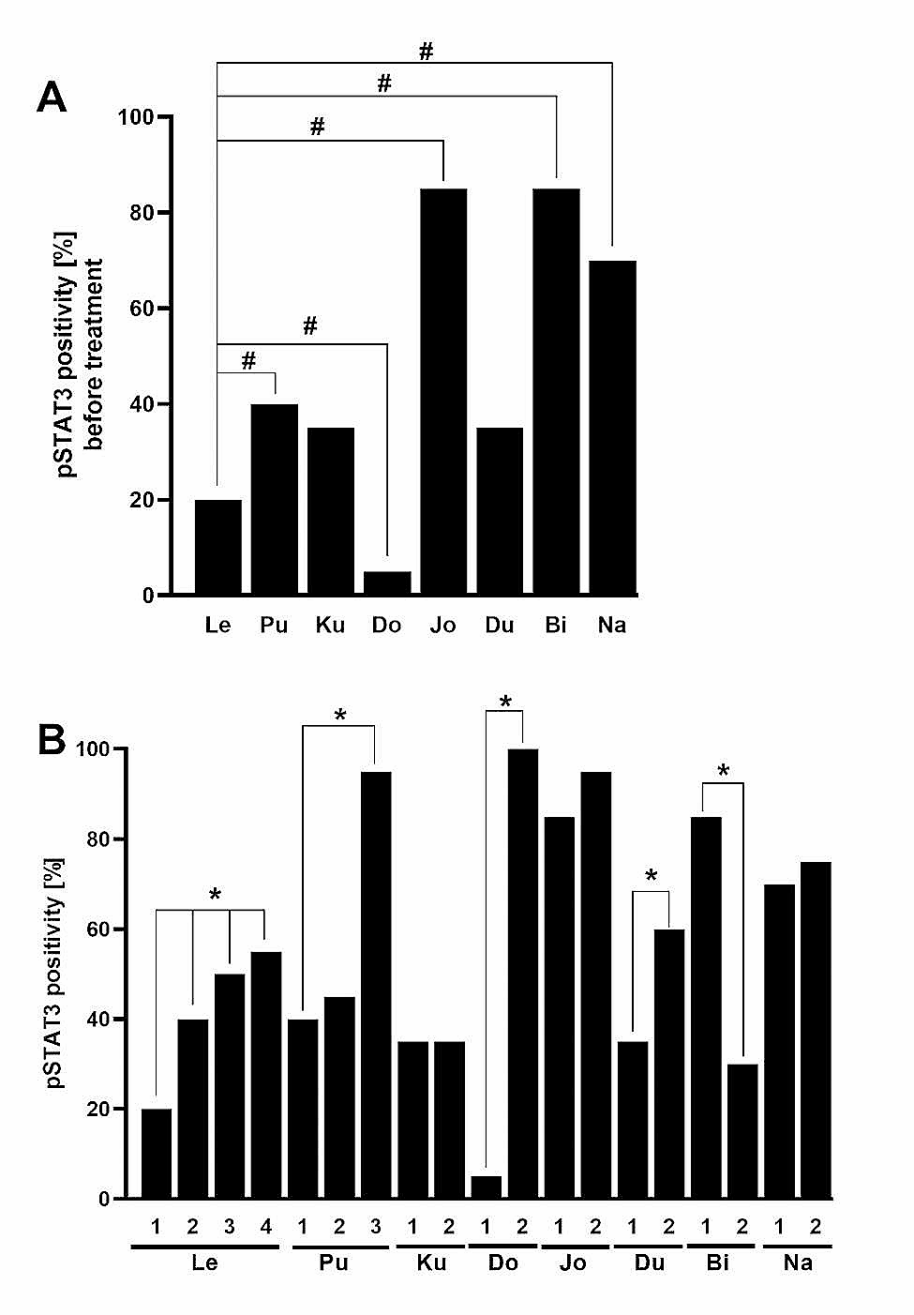
IHC analysis and quantification of pSTAT3 expressions in samples from repeatedly resected gliomas. pSTAT3 expressions in the first glioma sample of all patients (**A**), pSTAT3 expressions in reresected patients´ glioma samples (**B**). The bar number indicates the sequence of resections. *p ˂ 0.05 significant difference in expression of pSTAT3 between first and other resected samples of individual patients; #p ˂ 0.05 significant difference in expression of pSTAT3 between Le and further first resected samples from other investigated patients

IHC analysis was performed in all resected glioma samples where the presence and expression of tumor-specific, hypoxia-related and EMT markers were evaluated. MGMT promotor methylation positivity was detected in patients Do, Jo and Bi in all their resected tumors. A mutant form of IDH1 was found in patients Ku and Na, again in all tumors, however, molecularly, it was confirmed in Ku patient only. Expressions of Olig2, SOX11, vimentin, ZEB2 and TWIST decreased betweeen first and second resection in most of the investigated patients while an increased or the same level of CD44 at individual resections was noted. The most interesting trend was found in pSTAT3 expression. An elevated pSTAT3 level was observed with repeated glioma resections, except in patient Bi samples, where the trend was reversed (Table 2; Fig. 1). Collectively, obtained results from IHC analysis suggested that of all determined markers only pSTAT3 expression convincingly increased in reresected glioma samples in most analyzed patients.

### Mutation status of patients

Le patient, who did not undergo any radiotherapy or chemotherapy, had a PTEN mutation just in the 1st resected sample; the 2nd, 3rd and 4th samples were positive for wt PTEN. Opposite results were found in Pu patient– the mutant PTEN, IDH2 and TP53 were detected in the 2nd and 3rd samples only. PI3KCA was mutated in the 1st and 2nd samples of the patient Le while other two resections were PI3KCA wt (Supplementary Table 3). Mutation status of 9-glioblastoma related genes in all resected gliomas of individual patients showed a significant heterogeneity with no recognizable patterns and associations.

**Table 2:** IHC evaluation of the most common markers related with hypoxia in repeatedly resected gliomas

IHC analysis of hypoxia related markers																			
sample	resection	MGMT prom. methylation	EGFR amplified yes/no	ATRX - L = loss; *P* = positivity	p53	Olig2%	SOX10%	SOX11%	MEOX2%	CD44%	MERTK %	Vimentin %	ZEB1%	ZEB2%	TWIST %	HIF1a %	HIF1b %	STAT3%	pSTAT3%
**Le**	**1st**	no	no	L	mut	65	0	0	35	100	10	35	95	10	70	1	0	0	20
	**2nd**	no	no	*P*	mut	=	-	-	-	=	-	↑	-	↑	↓	-	↑	-	↑
	**3rd**	no	no	*P*	mut	↓	=	↑	↓	=	↓	↓	↓	↓	=	↑	↓	↑	↑
	**4th**	no	no	*P*	mut	↑	=	↑	↑	=	↑	↑	↑	↑	↑	↓	↑	↑	↑
**Pu**	**1st**	no	no	*P*	mut	95	0	10	95	95	-	100	-	100	100	0	60	100	40
	**2nd**	no	no	L	mut	↓	↑	↓	↓	↑	-	↓	75	=	↓	=	↓	-	↑
	**3rd**	no	no	*P*	mut	↑	↓	↑	↑	↑	27	↑	↑	=	↑	=	↑	=	↑
**Ku**	**1st**	no	no	*P*	wt	100	85	80	0	95	10	12	95	100	40	0	80	60	35
	**2nd**	no	no	*P*	wt	↓	↑	↓	=	↑	-	↓	↓	-	-	=	=	-	↑
**Do**	**1st**	yes	yes	L	wt	85	0	3	5	87	5	100	100	100	100	0	0	-	5
	**2nd**	yes	yes	*P*	wt	↓	=	↓	↑	↑	-	↓	=	-	=	=	↑	70	↑
**Jo**	**1st**	yes	yes	L	wt	40	0	0	15	100	5	95	35	100	0	0	15	-	85
	**2nd**	yes	yes	*P*	wt	↓	=	↑	↓	=	↑	↑	↑	=	↑	↑	-	50	↑
**Du**	**1st**	no	yes	*P*	wt	32	0	15	25	100	-	100	-	100	5	0	35	60	35
	**2nd**	no	yes	L	wt	↓	↑	↓	↓	=	5	↓	60	↓	=	=	↓	60	↑
**Bi**	**1st**	yes	no	*P*	mut	50	40	20	10	100	20	82	65	100	50	0	20	90	85
	**2nd**	yes	no	*P*	mut	↓	↓	↓	↓	=	↓	↓	↓	=	↓	↑	=	↓	↓
**Ná**	**1st**	no	no	L	wt	60	50	5	0	100	20	80	95	100	90	0	40	95	70
	**2nd**	no	no	L	wt	↓	↓	↓	=	=	↓	↓	↓	↓	↓	=	↓	↓	↑

### Primocultures, their chemosensitivity and expression of hypoxia-specific markers

Tumor tissues from repeated resections were also used for primary culture derivation using mechanical dissociation. Based on quality of derived cells, primary cells from samples of Le3 (GBMLe3), Le4 (GBMLe4) and Do2(GBMDo2) patients were cultivated and selected for further analyses.

Firstly, sensitivity to TMZ was tested in freshly derived cultures. All three tested cell lines widely varied in IC50 values, with most sensitive being GBMLe4 cells (5.5x more sensitive than GBMLe3 and almost 67x more sensitive than GBMDo2) (Table 3).

**Table 3:** Comparison of proliferation and IC50 values (µM) of temozolomide (TMZ) treatment (48 h exposition) in primary glioblastoma cells isolated from patient´s samples (GBM26, GBM43 and GBM59). Endpoint detection was performed using WST-1 analysis

glioblastoma cells	proliferation [%]	TMZ [µM]
GBMLe3	100.00 ± 3.06	2941.00
GBMLe4	514.98 ± 10.09	529.60
GBMDo2	230.58 ± 7.00	35435.00

**Fig. 2:**
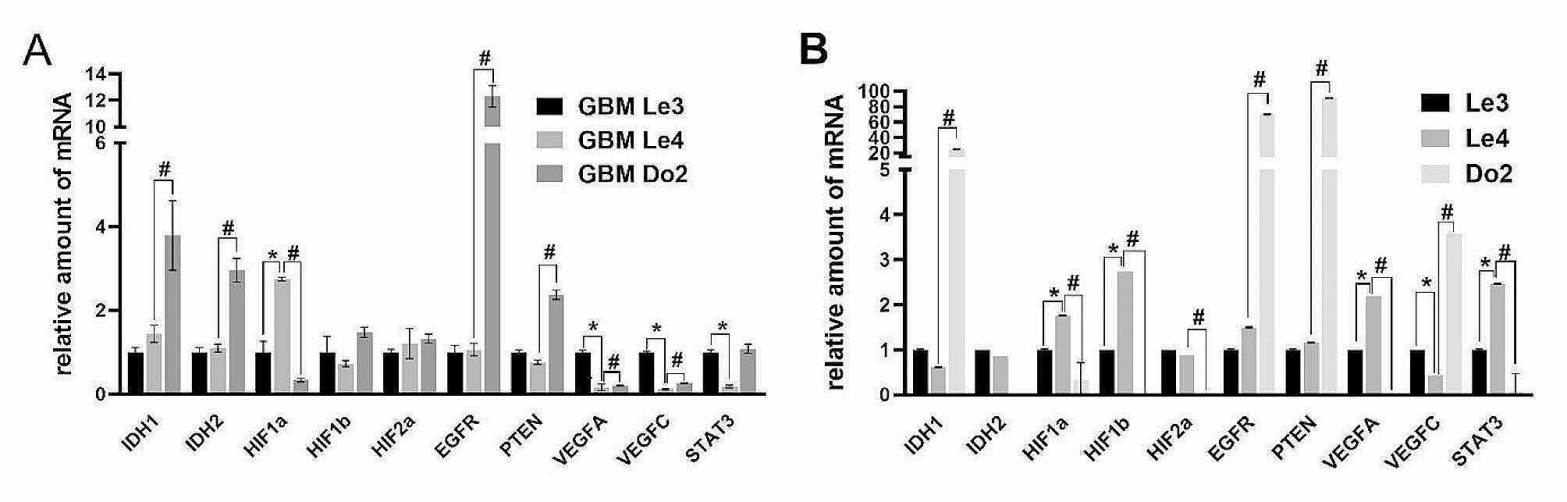
The expression of selected hypoxia-related markers (IDH1, IDH2, HIF1a, HIF1b, HIF2a, EGFR, PTEN, VEGFA, VEGFC and STAT3) in primary glioma cells GBMLe3, GBMLe4 and GBMDo2 (**A**) and in cryopreserved samples corresponding to the tumor used for particular primary glioma culture derivation (**B**) at mRNA level. The expression of mRNA was determined by RT-PCR. Data are expressed as fold increase ± SD of averages from two independent experiments. Beta-2-microglobulin was used as a housekeeping gene. * *p* < 0.05 GBM26 vs. GBM43; # *p* < 0.05 GBM26 vs. GBM59

Secondly, the expression of selected malignant glioma and hypoxia-specific markers was evaluated at mRNA level in both primary glioma cells (Fig. 2A) and corresponding cryopreserved tumor samples (Fig. 2B). Presented results illustrate the fact that the expression of most measured markers in individual cases displayed marked differences in relation to the model analyzed, i.e., primocultures versus cryopreserved samples of original tumor. Among notables exceptions are included expression of STAT3 in Le3, HIF1a in Le3, Le4 and Do2 as well as EGFR in Le3, Le4 and Do2.

### STAT-3 knockout and its effect on chemosensitivity and expression of hypoxia-related markers in vitro**and**in vivo

Since derived primary glioma cultures significantly differed in their sensitivity to TMZ as well as in expression profiles of examined glioma and hypoxia-related markers, verification experiments were carried out on stabilized cell lines U87MG and its STAT3 knockout variant U87MG-STAT3KO prepared by the CRISPR / Cas methodology (Supplementary Fig. 1). Results from viability testing indicated that compared to parent U87MG cells, their STAT3 KO variant showed much lower sensitivity to TMZ (Table 4).

**Table 4:** Comparison of IC50 values (µM) of temozolomide (TMZ) treatment (48 h exposition) in two stabilized glioma cells U87MG and its STAT3 KO variant. Endpoint detection was performed using WST-1 analysis

glioma cell line	Proliferation [%]	TMZ [µM]
U87MG IDH1wt	100.00 ± 14.13	3056.00
U87MG STAT3KO	132.37 ± 20.89	4138.00*

**Fig. 3:**
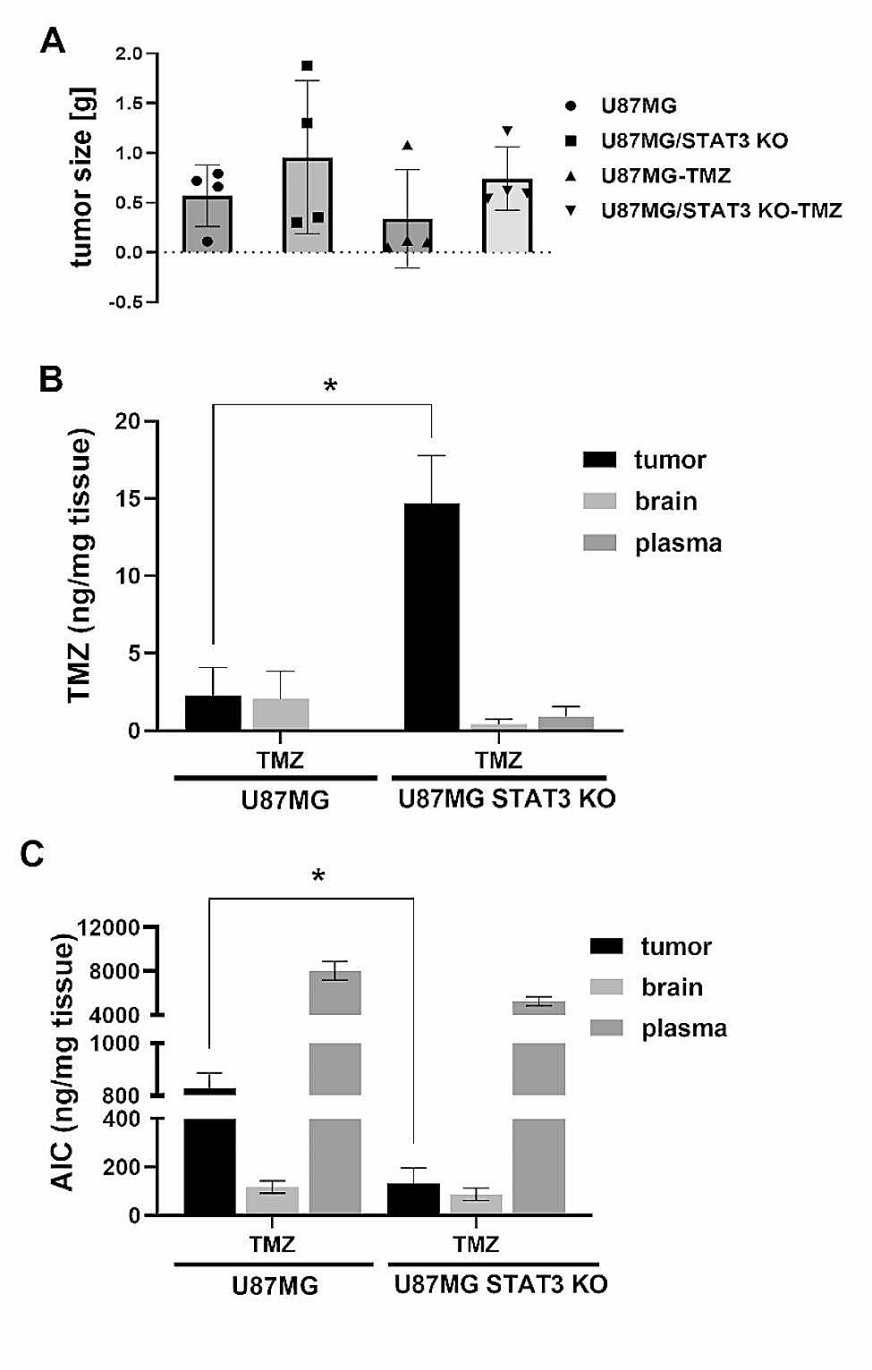
Comparison of tumor growth and drug accumulation in Foxn1-nu mice after implantation of glioma cell lines followed by TMZ treatment. Tumor size of implanted (*n* = 4) (**A**) U87MG IDH1wt, U87MG STAT3 KO with and without TMZ (0.9 mg/kg) treatment. Evaluation of accumulation of (**B**) TMZ and (**C**) its metabolites AIC inside the brain, tumor and plasma in tumor bearing mice with implanted U87MG IDH1wt, resp. U87MG STAT3 KO. The administration of drug (TMZ– 0.9 mg/kg) begins two weeks after implantation (from day 15. to day 28. daily). Organs were collected 15 min after last TMZ application. Confidence interval values of tumor size are shown as mean ± SD. The data of drug accumulation are expressed as ng per mg of tissue. Measurements were performed in two independent experiments

Both glioma cell lines (U87MG and its STAT3 knockout variant U87MG-STAT3KO) were next implanted into the Foxn1-nude mice and for 28 days the tumor growth was monitored with simultaneous administration (from 15th day after implantation) of TMZ at 0.9 mg/kg concentration. At the end of the experiment, the tumors were extracted, weighed and their size was measured. Compared to U87MG tumor group, an incresed tumor size was recorded in U87MG STAT3KO tumor bearing mice with an overall response to TMZ being also more robust in U87MG tumor bearing mice (Fig. 3A).

Tissue-specific distribution and concentration of TMZ and its metabolites (MTIC and AIC) were analyzed using LC-MS analysis in tumors, brain and plasma samples collected 15 min after last TMZ administration. Significanly higher TMZ concentration was detected in U87MG STAT3KO group in tumors. TMZ inactive metabolite AIC was found in a significantly lower amount in this tumor group. In any collected sample, active TMZ metabolite MTIC could not be determined (Fig. 3B and C). Results of this part of experiments showed that in tumors originating from U87MG cells with deleted STAT3 the parent TMZ accumulates to the high extent, but it is not seemingly converted to its active metabolite resulting in an uninhibited growth of tumor. Moreover, high levels of TMZ metabolite (AIC) in the plasma may indicate that TMZ is quickly systemically converted into a harmless product and thus fails to inhibit tumor growth.

**Fig. 4:**
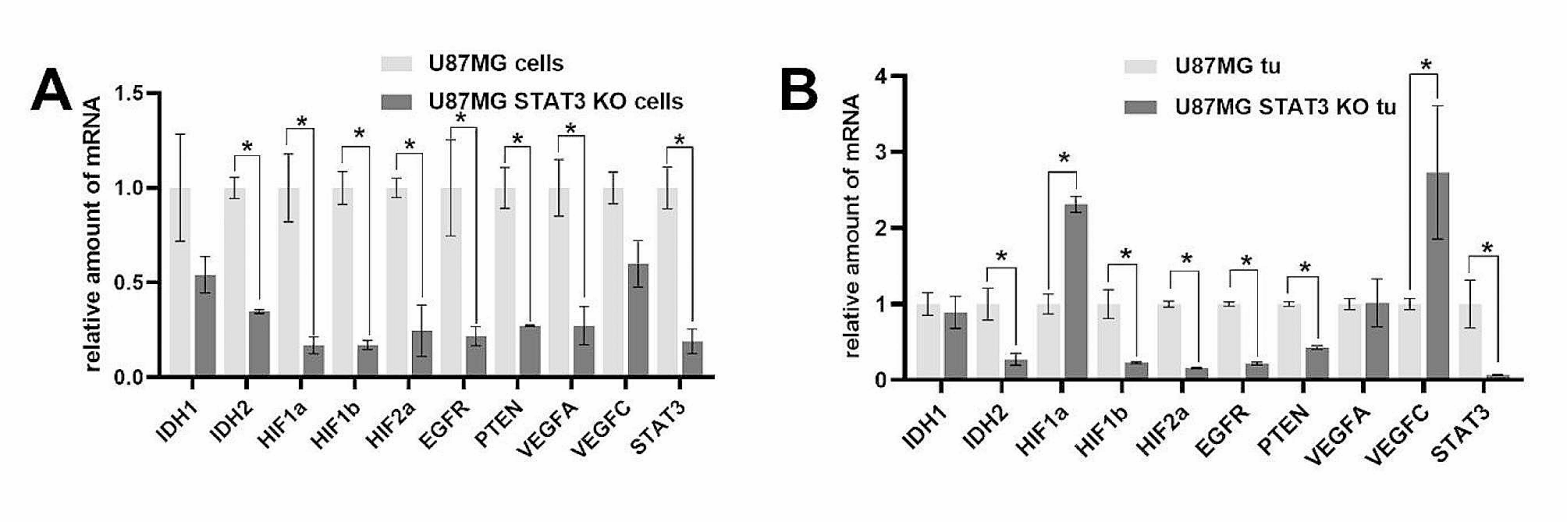
The expression of selected markers related with hypoxia (IDH1, IDH2, HIF1a, HIF1b, HIF2a, EGFR, PTEN, VEGFA, VEGFC and STAT3) in glioma U87MG and U87MG STAT3 KO glioma cell lines (**A**) and glioma samples collected from Foxn1-nu mice with implanted U87MG and U87MG STAT3 KO glioma cells on mRNA level (**B**). Tumors were collected 28 days after glioma cell implantation and processed as described in Materials and methods section

In glioma cell lines and collected tumor samples, the expression of glioma and hypoxia related markers on mRNA level was also examined. In glioma cell lines, the expression of all evaluated markers was downregulated in U87MG STAT3 KO variant compared to parent U87MG cells. In tumor samples, the expression of *IDH2, HIF1b, HIF2a, EGRF, PTEN* and *STAT3* was significantly downregulated, but *HIF1a* and *VEGFC* expressions were upregulated in U87MG STAT3 KO variant in comparison with U87MG cells (Fig. 4). Presented results indicate that deletion of STAT3 has a significantly suppressive effect on expresison of examined glioma and hypoxia related markers in vitro which could be recapitulated in vivo where, however, this effect is not universal as seen in case of *HIF1a* and *VEGFC* expressions.

## Discussion

Despite intensive investigations into the nature of glioblastoma which resulted in identification of many important mechanisms and driving forces, we are still lacking complete understanding of pathogenesis of this tumor. Moreover, and most importantly, crucial differences between standard patients and LTS patients are missing, to the large degree due to uncovered leads and incomplete evidence. Thus, the present study aimed to find out whether expression of select hypoxia markers and related signals would better characterize glioblastoma in a cohort of LTS patients.

Our initial results demonstrated mutational heterogeneity in select analyzed glioblastoma-related genes among included patients and in some cases in their serial tumor resections too. It concerned, among others, IDH1 status where samples of two of our patients carried IDH1mt variant, which has been previously suggested as the potential explanation of LTS in glioblastoma patients [19]. Still, this hypothesis could not have been proven in other studies [20]. Moreover, in our case the mentioned patients with IDH1mt did not survive significantly longer than others with IDH1wt, indicating that the present IDH1 variant alone is very unlikely a main contributor to LTS. These outcomes generally confirm and correspond to other studies which reported a highly variable and individual genetic terrain among LTS glioblastoma patients [21, 22]. Interestingly, chromosomal instability, the chief reason of genetic heterogeneity in tumors, is pervasive in high grade gliomas. Still, it is not considered to be a major contributor to the phenomenon of LTS in glioblastoma too although it has been proposed that specific chromosomal instability signatures might have some prognostic value in this type of malignancy [23].

Our subsequent evaluation of expression of targeted hypoxia, EMT and glioma-related markers revealed several opposing trends in individual analyzed marker groups. Still, a significantly increased expressions of STAT3 and pSTAT3 were found almost universally in repeated resections of most analyzed patients, thereby suggesting a possibly pronounced role of this molecule in recurrent glioblastoma. Accordingly, it is nowadays known that STAT3 is increasingly expressed in glioblastoma as shown by us and other groups [24, 25], and this expression has likely significant influence on several key biological characteristics of this tumor such as immunosuppressive microenvironment, hypoxia-induced angiogenesis and tumor cells migration [26, 27]. In addition, STAT3 was shown to be a positive regulator of HIF1α, VEGF, MMP-2 and *TWIST* in hypoxic glioblastoma [28] and its suppression induced proliferation arrest and apoptosis in glioblastoma cells [29]. To verify this observation in our analyzed patients, we have established primary glioblastoma cultures from their glioblastoma samples and compared several of their biological characteristics among them and with matched cryopreserved tumor samples. Resulting data indicated a significantly varying sensitivity of cultures to temozolomide (TMZ) and differently expressed hypoxia biomarkers including STAT3. Similar expression differences were also found between matching primocultures and cryopreserved tumor samples. Since the expression of STAT3 in primary cultures and matched cryopreserved tumor samples did not reflect the original observations, we have attempted to study it upon experimental conditions of its genetic deletion in a stabilized model glioblastoma cell line. STAT3 knockout in U87MG IDH wild type cells resulted in their lesser sensitivity to TMZ as compared to the same cells with wild type STAT3. Moreover, in vivo these cells produced larger tumors displaying lesser sensitivity to TMZ despite more significant accumulation of TMZ and lower presence of inactive TMZ metabolite. Conversely, loss of STAT3 suppressed expression of all evaluated hypoxia and EMT markers in glioblastoma cell line as well as in analyzed tumor originating from these cells with notable exception of *HIF1a* and *VEGFC*. Collectively, these results seem to suggest that loss of STAT3 at least in the present model does not render cells more chemosensitive to TMZ nor does it act suppressively on tumor growth in vivo. This finding is not consistent with the present understanding of the hypoxia-HIF1-STAT3-TMZ sensitivity axis and might thus relativize current efforts to develop specific STAT3 inhibitors to improve efficacy of glioblastoma management [30]. In this context, it must be verified whether the used technology of targeted STAT3 deletion did not produce some incidental off target effects in exposed cells with resulting lesser chemosensitivity to TMZ being only temporary or permanent. Consequently, larger panels of cell lines and matching in vivo experiments would be required. All the more so since it is necessary to mention here that our used U87MG cells are not considered an optimal model for glioblastoma studies due to their unclear origin [31, 32] which poses a certain limitation of our study. On the other hand, these cells are commonly employed in similar researches [33, 34] and thus it may argued that results obtained with them are not entirely biased. Finally, it is known that IL-6/JAK/STAT3 pathway is very plastic with a number of feedback loops and crosstalk points with other signaling systems which may then explain why knockout of STAT3 did not improve chemosensitivity or tumor growth both in vitro and in vivo as has been discussed in case of STAT3 inhibitors [30].

All together, data presented in this work illustrate complex expression patterns of select hypoxia and EMT-related markers in recurring glioblastoma samples as well as in cultures derived from them with no clear indefinable positive or negative correlations. It concerns for instance both HIF-1 and HIF-2 whose expression levels did not reflect a recurring nature of analyzed glioblastoma. It was also the case of STAT3 whose expression consistently increased in original tumor samples but not in derived primocultures or matched cryopreserved tumor specimens. In addition, subsequent studies on the role of STAT3 in the context of glioblastoma hypoxia demonstrated opposing effects of its deletion on cell viability as well as the expression of hypoxia and EMT markers. It is also possible that hypoxia and STAT3 operate in a larger tumor context and their individual roles are not sufficiently distinctive and standalone to universally characterize glioblastoma biology in LTSs in a similar manner to other analyzed markers, signatures or profiles. Another important factor to be taken into account is the nature and topography of hypoxia which is known to be spatially defined within a given glioblastoma but very likely heterogeneous across different tumors of individual patients. Its actual extent and distribution may significantly modulate local signaling events [35, 36] and thus its detailed mapping might identify patients with high hypoxia burden and verify whether this parameter associates with specific signaling and LTS status. Individually varied hypoxia extent could also be responsible for a discrepancy between HIF1 and pSTAT3 expression levels detected in particular analyzed samples which contradicts the commonly acknowledged expression patterns and functional relationship between both markers. On the other hand, since both HIF1 and STAT3 expressions were more correlated in primocultures and matching cryopreserved samples, one has to consider sensitivity and specificity of used detection techniques.

Hypoxia landscapes in individual glioblastoma samples also correlate with the presence of glioma stem cells [37] whose biological features such as non-proliferative status, specific signaling profiles and resistance likely influence glioblastoma resistance and aggressiveness and, consequently, patient survival. In our work we have not specifically focused on identifying glioma stem cells and their role in our cohort of glioblastoma LTS, however, the possible presence of such populations might represent a promising, hitherto unaddressed feature of glioblastoma which should be investigated in future.

Although results of this work did not reveal in recurrent glioblastoma samples generally unanimous and distinct factors characteristic for LTS patients, STAT3 and its expression appears to be a feasible point for evaluation of existence and roles of hypoxia in recurrent glioblastoma. It is further strengthened by the fact that in our Cox regression model STAT3 came out as a statistically significant marker of patient survival. These conclusions are nevertheless preliminary and limited by small patient/sample size. A larger confirmation of these results will be needed to further validate them.

### Electronic supplementary material

Below is the link to the electronic supplementary material.

Supplementary Material 1

## Data Availability

The datasets generated and/or analyzed during the current study are available in the [UNIPROT] repository, [accession number ID P40763].
