# Supplementary material for: Expression of STAT3 and hypoxia markers in long-term surviving malignant glioma patients
Source: BMC Cancer. 2024 Apr 23;24:509. doi: 10.1186/s12885-024-12221-w (PMC11036726; doi:10.1186/s12885-024-12221-w)
Supplement: Supplementary file 1 — Supplementary Material 1 [file 12885_2024_12221_MOESM1_ESM.docx]

**Supplementary files:**


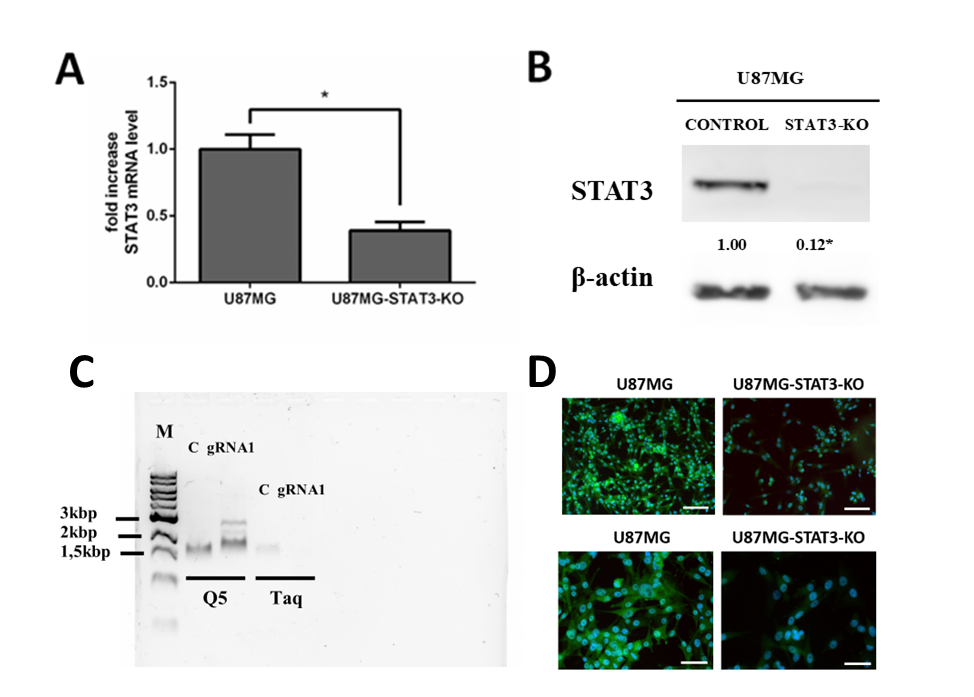


*Supplementary figure 1. – STAT3 knockout in cultivated GBM stabilized cell line U87MG. Cells were transfected using STAT3 Human Gene Knockout kit (with gRNA1) and knockout of STAT3 was verified in vitro on mRNA level (A) and protein level (B, D) as described in the Materials and Methods section. Furthermore, DNA evaluation of CRISPR-Cas9 transfection using PCR was carried out (C) as described in the Materials and Methods section *p ˂ 0.05 STAT3 expression in transfected vs non-transfected cells*

*M-marker; P1-P10 – subcultivation of U87MG cells after CRISPR-Cas9 transfection; P43 – control non-transfected U87MG cells; gRNA1 – U87MG transfected cells using gRNA1; gRNA2 – U87MG transfected cells using gRNA2*

*Fluorescence microscopy, magnification 200x (upper row), 400x (lower row). Scale 20µm.*


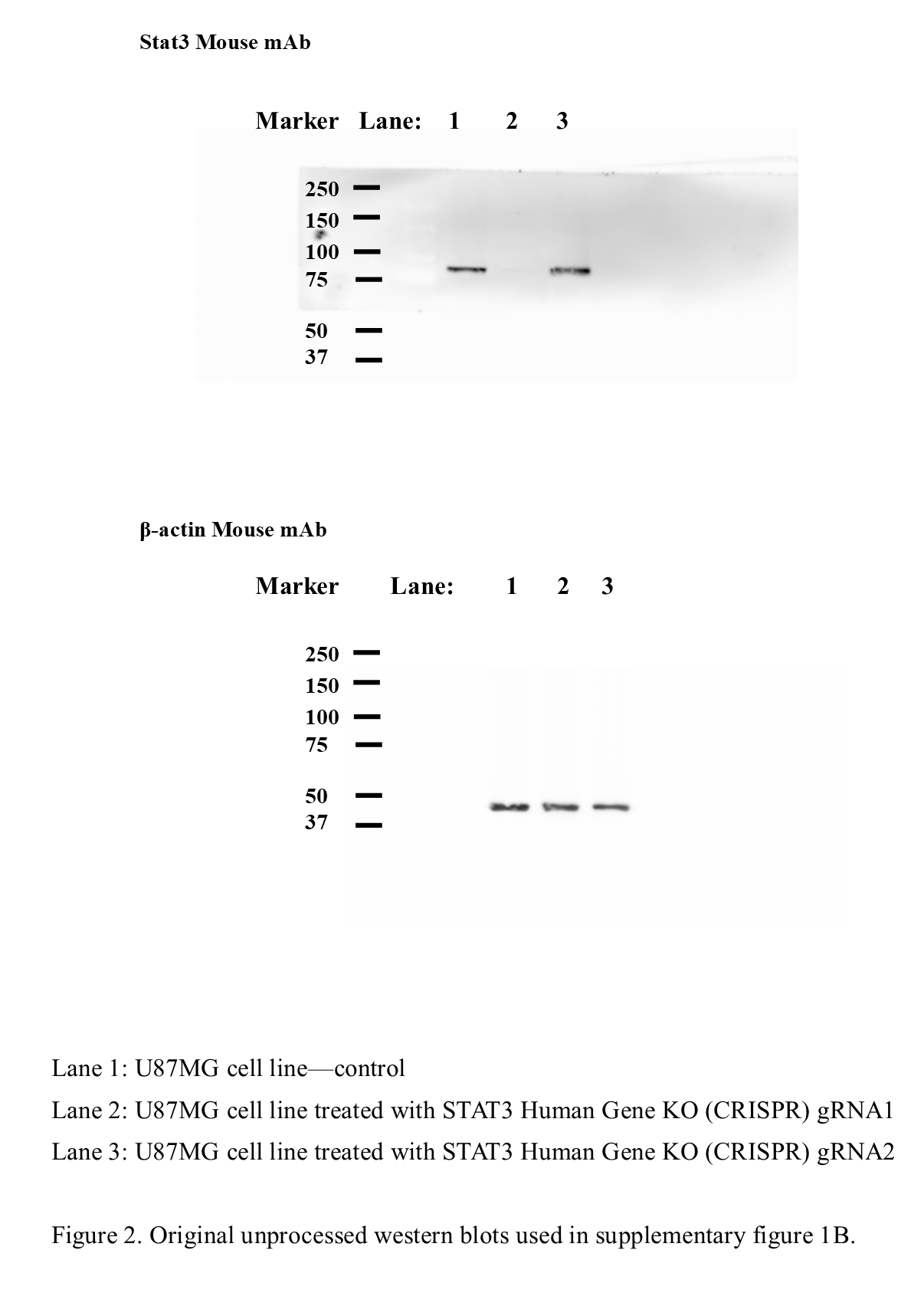


*Originals for supplementary figure 1B. – CRISPR-Cas9 mediated knockout of STAT3 expression in U87MG cell line. Beta actin expression was used as control. Original unprocessed immunoblots.*

*Lane 1: U87MG cell line - control*

*Lane 2: U87MG cell line treated with STAT3 Human Gene KO (CRISPR) gRNA1*

*Lane 3: U87MG cell line treated with STAT3 Human Gene KO (CRISPR) gRNA2*


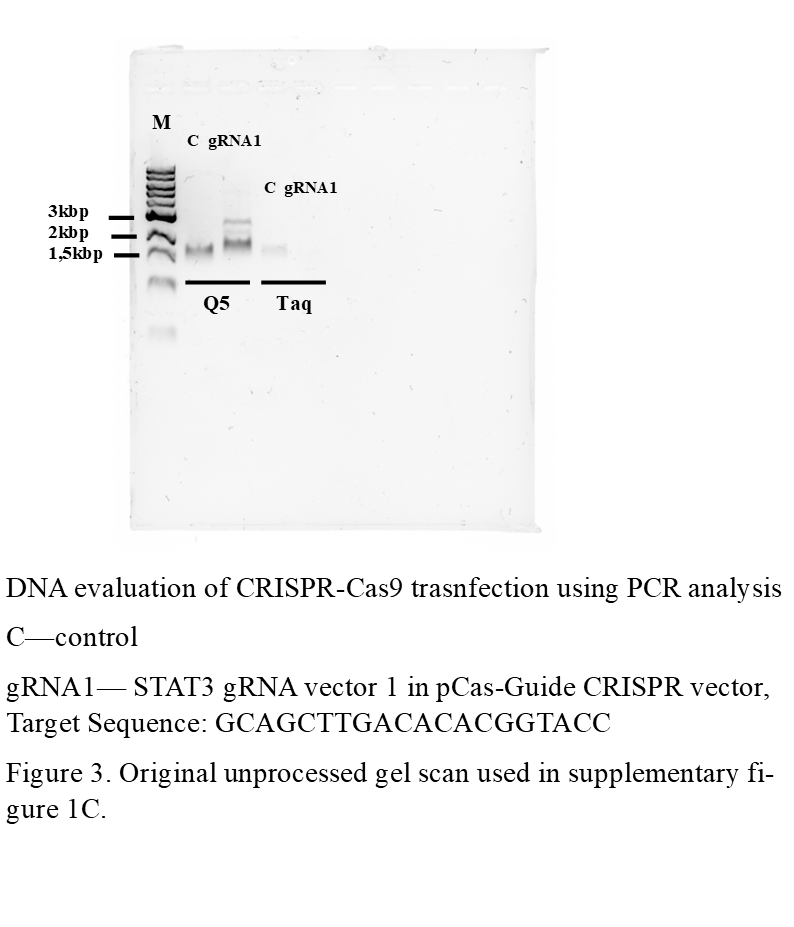


*Originals for supplementary figure 1C. – evaluation of CRISPR-Cas9 transfection efficiency at DNA level using PCR. Original unprocessed gel scan.*

*C – control*

*gRNA1 – STAT3 gRNA vector 1 in pCas-Guide CRISP vector.*

*Target Sequence: GCAGCTTGACACACGGTACC*

*
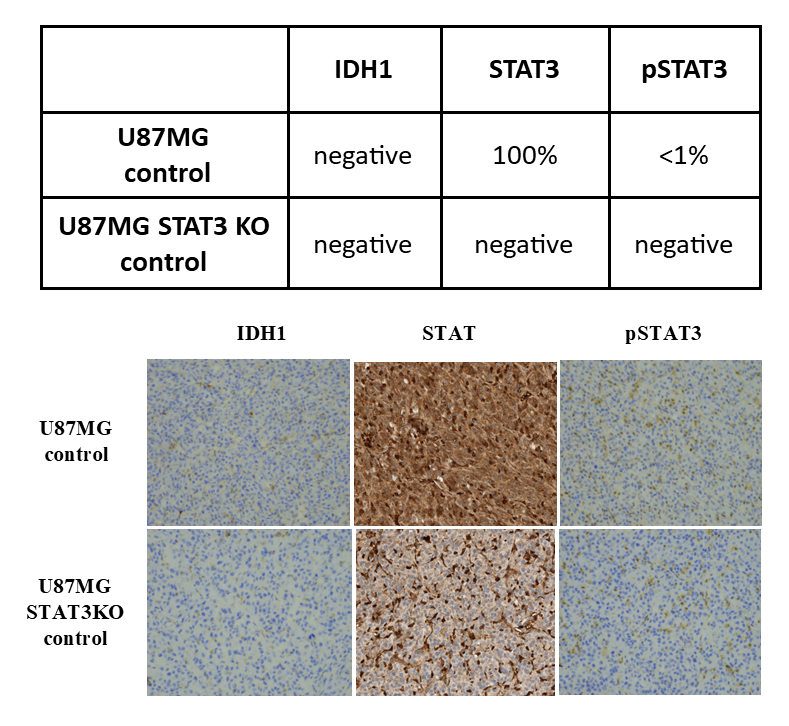
*

*Supplementary figure 2: Immunohistochemical analysis of the glioma sample collected from Foxn1-nu mice with implanted U87MG and U87MG STAT3 KO glioma cells collected from tumor-bearing mice U87MG IDH1wt and U87MG STAT3 KO in untreated mice. GBM samples were processed and IDH1, STAT3, pSTAT3, were detected by immunohistochemistry and histochemistry, as described in the Materials and Methods section.*

*Supplementary table 1* – Antibodies and details of immunohistochemical reactions used in the study

| Antibody | Clone | Dilution | Manufacturer | Positive control |
| --- | --- | --- | --- | --- |
| GFAP | EP672Y | RTU | Ventana, Basel, Switzerland | Brain |
| MEOX2 | 6A5 | 1:1000 | Sigma Aldrich s.r.o., Prague, CR | Appendix – HEV* |
| EGFR | 31G7 | 1:100 | Novus Biologicals, Ontario, Canada | Placenta |
| SOX11 | MRQ-58 | 1:50 | Cell Marque, Rocklin, CA, USA | Foetal brain |
| SOX10 | EP268 | 1:50 | Cell Marque, Rocklin, CA, USA | Appendix |
| PDGFRa | C-9 | 1:100 | SCBT Inc., Dallas, TX, USA | Appendix |
| MerTK | Y323 | 1:400 | Abcam, Cambridge, MA, USA | Appendix |
| CD44 | MRQ-13 | 1:400 | Cell Marque, Rocklin, CA, USA | Brain |
| CD56 | MRQ-42 | 1:2000 | Cell Marque, Rocklin, CA, USA | Appendix |
| CD57 | TBO1 | 1:50 | Dako, Glostrup, Denmark | Appendix |
| Olig2 | EP112 | 1:200 | Cell Marque, Rocklin, CA, USA | Brain |
| IDH1 R132H | H09 | 1:100 | Dianova GmBH, Hamburg, Germany | Glioma |
| p53 | DO7 | RTU | Ventana, Basel, Switzerland | Breast carcinoma |
| Carbon anhydrase 9 (CAIX) | EP161 | 1:100 | Cell Marque, Rocklin, CA, USA | Renal carcinoma |
| ZEB1 | CL0151 | 1:100 | Novus Biologicals, Ontario, Canada | Appendix |
| ZEB2 | polyclonal | 1:50 | Thermo Fisher, Brno, Czech Republic | Appendix |
| TWIST | 10E4E6 | 1:500 | LS Bio, Lynnwood, WA, USA | Placenta |
| SNAI1 | polyclonal | 1:200 | Merck KGaA, Darmstadt, Germany | Kidney |
| STAT3 | 124H6 | 1:400 | Cell Signalling, Danvers, MA, USA | Parathyroid gland |
| STAT3 p705 | D3A7 | 1:100 | Cell Signalling, Danvers, MA, USA | Parathyroid gland |
| HIF1a | EP118 | 1:50 | BioSB, Santa Barbara, CA, USA | Placenta |
| HIF1b | D28F3 | 1.50 | Cell Signalling, Danvers, MA, USA | Placenta |
| CD34 | QBEnd10 | 1:50 | Cell Marque, Rocklin, CA, USA | Appendix |

## * HEV – high endothelial venules

*Supplementary table 2* – Primers used in the study

| GCACACAGTTCCTTCCAAATG | h_IDH1_f |
| --- | --- |
| ACGCCCATCATATTTCTTCAGA | h_IDH1_r |
| CGTGTGGAAGAGTTCAAGCT | h_IDH2_f |
| GGGACTAGGCGTGGGATG | h_IDH2_r |
| TGTACCCTAACTAGCCGAGG | h_HIF1a_f |
| ATACGTGAATGTGGCCTGTG | h_HIF1a_r |
| TGTGGACCCAGTTTCTGTGA | h_HIF1b_f |
| GCCAAGTCCATTCCTGCATC | h_HIF1b_r |
| GCCCAATAGCCCTGAAGACT | h_HIF2a_f |
| GTCCAGCTCATTGAAATCCGT | h_HIF2a_r |
| GCCACCCATATGTACCATCG | h_EGFR_f |
| TGCGTCTATCATCCAGCACT | h_EGFR_r |
| CAGTGGCGGAACTTGCAAT | h_PTEN_f |
| TCACCTTTAGCTGGCAGACC | h_PTEN_r |
| GAGAATCTCCAGGATGACTTTGA | h_STAT3_f |
| TTGCATGTCTCCTTGACTCTTG | h_STAT3_r |
| CTGGAGGAGCAGTTACGGTC | h_VEGFC_f |
| TCCTTTCCTTAGCTGACACTTGT | h_VEGFC_r |
| GCGGATCAAACCTCACCAAG | h_VEGFA_f |
| GCTCTATCTTTCTTTGGTCTGCA | h_VEGFA_r |
| TGCTGTCTCCATGTTTGATGTATC | h_B2M_f |
| TCTCTGCTCCCCACCTCTAAG | h_B2M_f |

*Supplementary table 3* – Mutational analysis of the most common markers related with hypoxia in repeatedly resected gliomas. All changes of interest in mutational status of analyzed targets are highlighted in bold

| **mutational analysis of hypoxia-related markers** | | | | | | | | | | | |
| --- | --- | --- | --- | --- | --- | --- | --- | --- | --- | --- | --- |
|  | **resection** | **IDH1** | **IDH2** | **PIK3CA** | **EGFR** | **PTEN** | **TP53** | **prom TERT** | **BRAF** | **RB1** |  |
| **Le** | **1^st^** | wt | wt | **mut (8%)** | wt | **mut** | **mut** | wt | wt | **mut** |  |
|  | **2^nd^** | wt | wt | **mut (24%)** | wt | **wt** | **mut** | wt | wt | **mut** |  |
|  | **3^rd^** | wt | wt | wt | wt | **wt** | **mut** | wt | wt | **mut** |  |
|  | **4^th^** | wt | wt | wt | wt | **wt** | **mut** | wt | wt | **mut** |  |
| **Pu** | **1^st^** | wt | **wt** | wt | wt | **wt** | **wt** | wt | wt | wt |  |
|  | **2^nd^** | wt | **mut** | wt | wt | **mut** | **mut** | wt | wt | wt |  |
|  | **3^rd^** | wt | **mut** | wt | wt | **mut** | **mut** | wt | wt | wt |  |
| **Ku** | **1^st^** | **mut** | wt | wt | wt | wt | wt | wt | **mut** | wt |  |
|  | **2^nd^** | **mut** | wt | wt | wt | wt | wt | wt | **mut** | wt |  |
| **Do** | **1^st^** | wt | wt | wt | **wt** | wt | wt | **mut** | wt | wt |  |
|  | **2^nd^** | wt | wt | wt | **mut** | wt | wt | **mut** | wt | wt |  |
| **Jo** | **1^st^** | wt | wt | wt | **mut** | wt | wt | **mut** | wt | wt |  |
|  | **2^nd^** | wt | wt | wt | **mut** | wt | wt | **mut** | wt | wt |  |
| **Bi** | **1^st^** | wt | wt | wt | wt | wt | **mut** | **mut** | wt | wt |  |
|  | **2^nd^** | wt | wt | wt | wt | wt | **mut** | **mut** | wt | wt |  |
| **Ná** | **1^st^** | wt | wt | wt | wt | wt | wt | wt | wt | wt |  |
|  | **2^nd^** | wt | wt | wt | wt | wt | wt | wt | wt | wt |  |
